# Larval and adult diet affect phenotypic plasticity in thermal tolerance of the marula fly, *Ceratitis cosyra* (Walker) (Diptera: Tephritidae)

**DOI:** 10.3389/finsc.2023.1122161

**Published:** 2023-03-28

**Authors:** Dylan A. Pullock, Kévin Malod, Aruna Manrakhan, Christopher W. Weldon

**Affiliations:** ^1^Department of Zoology and Entomology, University of Pretoria, Pretoria, South Africa; ^2^Citrus Research International, Mbombela, South Africa; ^3^Department of Conservation Ecology and Entomology, Faculty of AgriSciences, Stellenbosch University, Stellenbosch, South Africa

**Keywords:** fruit fly, nutrition, temperature, dietary compensation, thermal tolerance

## Abstract

**Introduction:**

Temperature fluctuations are important for the distribution and survival of insects. Rapid hardening, a type of phenotypic plasticity, is an adaptation that can help individuals better tolerate lethal temperatures because of earlier exposure to a sublethal but stressful temperature. Nutrition and sex are also known to influence a species ability to tolerate thermal stress. This study determined the effects of larval diet, adult diet, sex and hardening on the thermal tolerance of *Ceratitis cosyra* (Walker) (Diptera: Tephritidae) at lower and upper lethal temperatures.

**Methods:**

Larvae were raised on either an 8% torula yeast (high) or a 1% torula yeast (low) larval diet and then introduced to one of three dietary regimes as adults for thermal tolerance and hardening assays: no adult diet, sugar only, or sugar and hydrolysed yeast diet. Flies of known weight were then either heat- or cold-hardened for 2 hours before being exposed to a potentially lethal high or low temperature, respectively.

**Results:**

Both nutrition and hardening as well as their interaction affected *C. cosyra* tolerance of stressful temperatures. However, this interaction was dependent on the type of stress, with nutrient restriction and possible adult dietary compensation resulting in improved cold temperature resistance only.

**Discussion:**

The ability of the insect to both compensate for a low protein larval diet and undergo rapid cold hardening after a brief exposure to sublethal cold temperatures even when both the larva and the subsequent adult fed on low protein diets indicates that *C. cosyra* have a better chance of survival in environments with extreme temperature variability, particularly at low temperatures. However, there appears to be limitations to the ability of *C. cosyra* to cold harden and the species may be more at risk from long term chronic effects than from any exposure to acute thermal stress.

## Introduction

The impact of climate change on organisms is significant but differs depending on geographical region (1, 2). There is evidence to suggest that climate change increases the frequency of climate extremes such as unusually strong heatwaves and storms (3). These increases in extremes also change seasonally (2, 4). The resulting spatial and temporal diversity of weather events results in new or different climates, especially in terms of rainfall and temperature (5–7). Temperature fluctuations and the ability to adapt to or move in accordance with local changing temperature ranges plays an important role in global diversity patterns, metabolic strategies (such as ectothermy) and the distribution of species (8, 9). Insects, being ectothermic, are vulnerable to environmental temperature and its fluctuation as their physiological processes are temperature sensitive (10, 11).

One of the adaptations to better withstand local temperature fluctuations is rapid hardening, a type of phenotypic plasticity, which can either be rapid heat hardening at high temperatures or rapid cold hardening at low temperatures (12, 13). Rapid hardening occurs when an organism is exposed to a stressful non-lethal temperature for a short period of time, resulting in an increase in thermal tolerance, which may improve the ability of an individual to survive exposure to extreme temperatures that would otherwise be lethal (12). Rapid heat and cold hardening has been shown to improve the survival of a few insect species when exposed to extreme temperatures, both in a laboratory setting as well as in the field (14–18). Alongside the above-mentioned role in species distribution, this improvement in survival may also contribute to the invasiveness of pest species such as the Mediterranean fruit fly, *Ceratitis capitata* (Wiedemann) (Diptera: Tephritidae) (13), or invasive termite species like *Mastotermes darwiniensis* (Froggatt) (Blattodea: Mastotermitidae) (11).

Nutrition, like rapid hardening, is known to play a role in how a species survives at different high or low temperature extremes (10, 19–22). Andersen et al. (19) found that *Drosophila melanogaster* (Meigen) (Diptera: Drosophilidae) raised on a protein rich larval diet survived exposure to high temperatures better than those raised on a carbohydrate rich larval diet. In contrast, flies raised on the carbohydrate rich diet survived exposure to low temperatures better than their protein fed counterparts (19). Mitchell et al. (22) withheld protein as a food source to adult *C. capitata* and found a positive association between nutrient restriction and heat tolerance. The study by Andersen et al. (19) also showed that sex may influence the interaction between nutrition and thermal tolerance, but it should be noted that sex had no significant effect on thermal tolerance in some *Ceratitis* species (13). Poor nutrition at the larval stage of a holometabolous insect can be compensated for by selective feeding on a nutrient rich diet at the adult stage (23–28). Depending on species and other intrinsic factors, such dietary compensation would impact life history traits and responses to abiotic stress (29).

The marula fly, *Ceratitis cosyra* (Walker) (Diptera: Tephritidae), is a fruit fly species of particular interest because it is a sub-Saharan Afrotropical pest of phytosanitary concern that is known to negatively affect mango, *Mangifera indica* (Linnaeus) (Sapindales: Anacardiaceae) production (30–32). Apart from utilizing mango at the larval stage, *C. cosyra* also utilizes other fruit types including marula, *Sclerocarya birrea* ((A. Rich) Hochst) (Sapindales: Anacardiaceae); avocado, *Persea americana* (Miller) (Laurales: Lauraceae); peach, *Prunus persica* ((Linneaus) Batsch) (Rosales: Rosaceae); and guava, *Psidium guajava* (Linneaus) (Myrtales: Myrtaceae) (33). These fruit types vary in their nutritional contents, more specifically in their protein contents (34–37). Besides an adventive population of *C. cosyra* in the Indian Ocean island of Madagascar, the species is restricted to continental Africa (38). In a prediction of the potential distribution of *Ceratitis* pest species worldwide and in China, areas modelled as suitable for *C. cosyra* were predominantly subtropical (39). In these models, changes in global climatic conditions were not considered. Information on the thermal tolerance of *C. cosyra* would improve prediction of the potential distribution of the pest in a changing climate. However, with nutrition and rapid hardening potentially changing the thermal tolerance of insects, it is important to evaluate these interacting effects on the stress resistance phenotype of *C. cosyra*.

The aim of this study is to investigate the effect of larval diet, adult diet, sex and hardening on the thermal tolerance of *C. cosyra*, measured as lower and upper lethal temperatures. The flies are predicted to exhibit both rapid heat and rapid cold hardening as both have been observed in other *Ceratitis* fruit flies. Adult diet is also expected to influence thermal tolerance, with a carbohydrate rich diet improving survival at low temperatures and a protein rich diet improving survival at high temperatures. Sex is not expected to have any significant effect on thermal tolerance for this species.

## Materials and methods

### Maintenance and handling of flies

Flies were sourced from a culture held in the Department of Zoology and Entomology, University of Pretoria, South Africa. This culture was built using wild flies collected from marula fruit found in the northern parts of Tshwane Metropolitan Municipality, Gauteng province, South Africa. The culture was refreshed with newly collected wild flies annually by mating wild males reared from marula fruit with females from the established culture. The culture was housed in nylon mesh cages (32.5 × 32.5 × 32.5 cm, BugDorm-4F3030, MegaView Science Co., Taiwan), provided with separate dishes of sugar and yeast hydrolysate (HG000BX6.500, Merck, Wadesville, South Africa) as food sources as well as a cup of soaked cotton wool as a water source. Cultures were kept in a climate room at an average temperature of 22 ± 1°C at a relative humidity of 40-60% with a 12 hour light and dark cycle with dusk and dawn simulated where the lights turn on at 06:00 h (South Africa Standard time; GMT +2) and off at 18:00 h (28).

Eggs were collected using 100 ml plastic cups containing damp tissue paper and 3 ml of guava concentrate (Halls; Tiger Consumer Brands Limited, Bryanston, South Africa). The egg collection cups were covered with fine insect screen and then a double layer of laboratory film (Parafilm M, Bemis Flexible Packaging, Neenah, WI, USA) perforated with a pin. Both the insect screen and laboratory film were held in place by separate rubber bands. Females were given the opportunity to oviposit in these containers for 6-8 hours. Using distilled water, the eggs were rinsed off the mesh, the laboratory film and the cup into a flat-bottomed glass dish where they were allowed to settle. A 3 ml disposable Pasteur pipette was used to suck up the eggs and given time to settle at the pipette tip. Fly eggs were applied to the larval diet (described below) at a rate of 2.5 eggs/ml diet. The quantity of larval diet and eggs were varied to obtain the required number of adults for testing.

### Experiment 1. Determination of sublethal and lethal temperatures

**Figure 1:**
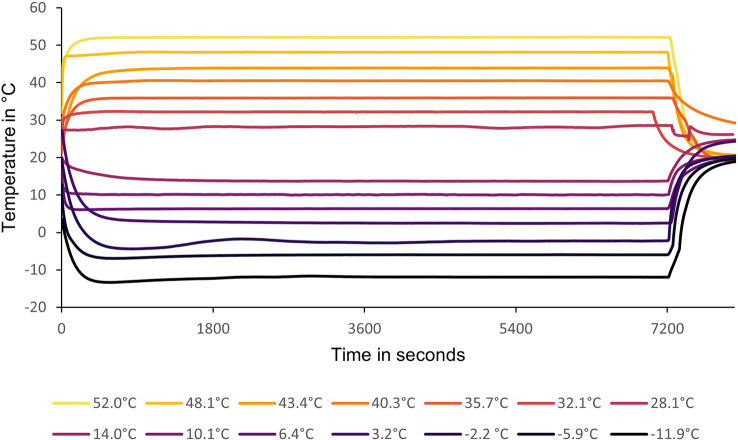
Upper and lower test temperatures recorded per second over a period of 7200 seconds or 2 hours in each test. The starting temperatures were the readings taken in the vials inside the water bath during each test. All replicates were subjected to the relevant test temperature at the same time.

Fly eggs were collected from the culture and placed on standard 8% torula yeast larval diet (Citrus Research International, Nelspruit, South Africa). Approximately 10 cups of larval diet were used to get the necessary flies for the preliminary thermal limit testing as well as enough excess to start a new culture with which to do hardening and thermal tolerance testing (Experiment 2). The temperatures planned for the test ranged from 28 to 52°C, increasing in increments of 4°C, for upper lethal temperature (ULT), and 14 to -10°C, decreasing in increments of 4°C, for lower lethal temperature (LLT). The actual test temperatures were averaged from thermocouple readings and are presented in **Figure 1**. Flies were tested at peak emergence (1-2 days after first adult emergence) and allowed to become accustomed to the vials for 30 minutes at 25°C before testing. Ten transparent plastic vials, each containing a group of 10 flies (five vials for males and five vials for females), were used to test each temperature using a programmable water bath (Model: K25-cc-NR, huber, Germany) with a 1:1 propylene glycol-water mix. Each vial represents a replicate. The different vial sets were exposed to each of the test temperatures for 2 hours. They were then checked at 1-, 2- and 24-hour periods after temperature exposure to determine the best recovery time for future use. Vials took roughly 10 minutes to reach test temperature in the water bath (**Figure 1**). Due to the numbers needed, multiple different egg groups were collected a day or two apart so that there would be enough male and female flies of the same age to run roughly two temp tests a day using the one water bath. Temperature test order was randomized. Survival was defined as normal behavior (locomotion or sustained movement) or reaction to mild external stimuli such as rolling the test vial in a hand or prodding with a pencil. The observations were recorded for analysis to calculate fly survival %, and to determine lethal (temperature causing only 50% survival) and lower and upper sublethal temperatures (most extreme temperatures where survival was still 100%). The calculated upper sublethal temperature was found to be within 1°C of the calculated ULT making its use impractical in Experiment 2. A sublethal temperature of 35°C was used instead due to its use in a study looking at two other *Ceratitis* species, *C. rosa* (Karsch) (Diptera: Tephritidae) and *C. capitata* (13). The relative humidity in the vials for Experiment 1 and 2 were calculated using a modified formula and are included in **Appendix 1**.

### Experiment 2. Larval and adult dietary effects on thermal tolerance

A standard 8% torula yeast diet (Citrus Research International, Nelspruit, South Africa) and 1% torula yeast diet (Citrus Research International, Nelspruit, South Africa) were used as the high and low protein larval diet based on the dietary trial results of Weldon et al. (28). In comparison, the protein content of marula fruit (*Sclerocarya birrea*) and mango (*Mangifera indica*), two known *C. cosyra* hosts, is 0.5% and 0.2% respectively (28, 40). Each larval diet was made by mixing 200 ml boiling water with 100 g powder (either 8% or 1% torula yeast) to form enough smooth paste to fill two 100 ml cups for use over two days after cooling. Enough larval dietary cups were inoculated with fly eggs to provide the number of flies needed to run the relevant thermal tests for all dietary combinations as well as extra flies used, before stress, to determine body weight. The incubation temperature was similar to those used in the maintenance of the fly cultures. Body weight data was collected using 10 individuals of each category, placed individually into their own microcentrifuge tubes of known weight. The difference between the weight of the fly in a tube and the tube alone was recorded as fly weight. Adults developed from each diet were exposed to three dietary regimes: none (i.e., no food), sugar and yeast hydrolysate (Su+YH) and just sugar (Su). Adults from each of the adult dietary regime were then subjected to hardening and thermal tolerance assays. Adults with no food were those that were collected at the peak of adult emergence (1-2 days after first adult emergence). Adults on either Su+YH or Su were left to feed for a period of 10 days. A source of water was also provided in each adult dietary regime. Adults of each dietary regime were exposed to hardening and thermal tolerance assays. Before assays, flies of each regime were kept in a cage and steps were taken to reduce crowding stress by limiting fly numbers to roughly 300 individuals in each cage.

The hardening and thermal tolerance assays were run using protocols adapted from Terblanche et al. (41). To ascertain whether *C. cosyra* rapidly heat or cold harden (RHH and RCH), 20 plastic vials (10 vials of males and 10 vials of females) each containing 10 individuals, were left to rest at 25°C for 30 minutes. Half of the vials for each sex were exposed to sublethal temperatures for 2 hours, thus hardening them. The remaining half of the vials were left to rest at 25°C for the same 2 hours so that they remained not hardened. All vials were then subjected to the relevant lethal temperatures (high or low). Incubators (Model: LTIE, Labcon, Maraisburg, South Africa) set at calculated sublethal temperatures were used for hardening treatments and a programmable water bath set at calculated lethal temperatures was used for thermal tolerance assays. This was done for all six dietary combinations (low protein larval diet/none, low protein larval diet/Su+YH, low protein larval diet/Su, high protein larval diet/none, high protein larval diet/Su+YH and high protein larval diet/Su) at high as well as low sublethal and lethal temperatures, and a second replicate was done with new flies to increase data for analysis and account for variability between batches. After exposure to potentially lethal temperatures, flies were left to recover for 24 hours (based on the results of Experiment 1) before survival was assessed in each vial, as in Experiment 1.

### Data analysis

Data analyses were performed in R version 3.6.1 using RStudio version 1.2.1335 (42). Data from low and high temperature exposures were analysed for Experiment 1 using separate generalized linear models (GLZ) with binomial error distribution to determine the effects of temperature and sex on fly survival. This was done using the number of flies alive and dead in each vial as dependent variables paired for analysis using the ‘cbind’ function in R, then the results were visualized using survival plots. The upper and lower lethal temperatures (LT_50_) and sublethal temperatures (ST_100_) were calculated from the model fit. The temperatures leading to other levels of proportional survival were also calculated for comparison with species tested in other studies. Stepwise deletion of each variable, and any interactions between them, as well as Akaike’s information criterion (AIC) were used to determine the minimal adequate models. A quasibinomial error distribution was used to account for overdispersion when needed. The LT_50_ and ST_100_ were calculated respectively using the ‘dose.p’ function, where p = 0.500 (50% fly survival) and p = 0.999 (close to 100% fly survival).

Data analyses for Experiment 2 were done using separate GLZs with binomial error distribution to determine the nested effects of larval diet, adult diet, hardening and sex as well as their interactions on fly survival at high and low test temperatures. Each model was generated using the ‘glm’ function as the variance of the random effect was null. A general linear model (GLM) was also used to determine the nested effects of diet, sex, and hardening, as well as their interactions, on the body weight of flies. Significant differences in the nested effects of the variables were determined using ANOVA tests. *Post hoc* Tukey tests were not run due to the complex nature of the nested data.

## Results

### Experiment 1. Determination of recovery time, sublethal and lethal temperatures

**Figure 2:**
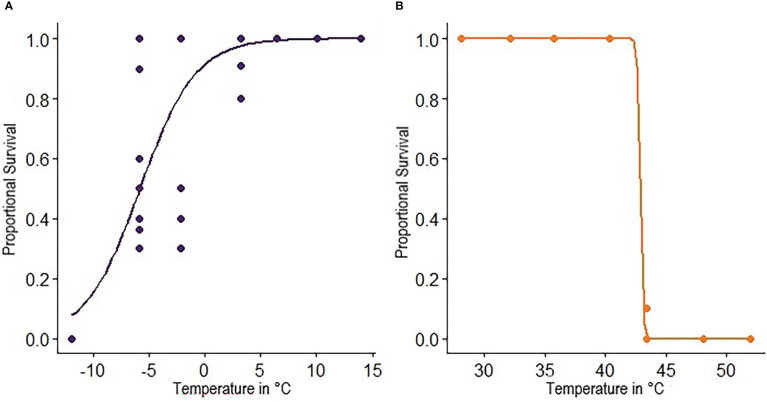
Proportional survival of *C*. *cosyra* as a function of test temperature. The LT_50_ is taken at a proportional survival of 0.5 and ST_100_ is taken at a proportional survival of 0.999. Test temperatures were **(A)** -11.9°C, -5.9°C, -2.2°C, 3.2°C, 6.4°C, 10.1°C, 14.0°C and **(B)** 28.1°C, 32.1°C, 35.7°C, 40.3°C, 43.4°C, 48.1°C, 52.0°C. Colored circles represent observed values, while lines show the inferred model.

Fly survival was greatest after a 24-hour recovery period, so this period of time was used for analyses of results from Experiment 1 and was the only recovery time used in Experiment 2. Only temperature was retained in the minimal adequate model and had a significant effect on survival when considering low temperatures (χ^2^ = 201.84, df = 1, p < 0.001) and high temperatures (χ^2^ = 964.89, df = 1, p < 0.001). Survival exhibited a sigmoidal relationship with temperature, with 100% mortality at -12°C and 100% survival at 6°C and above (**Figure 2**). In contrast, there was a precipitous drop in survival at test temperatures greater than 42°C (**Figure 2**). The lower and upper LT_50_ were calculated at -5.8°C and 42.9°C respectively while lower and upper ST_100_ were 10.3°C and 42.1°C (**Table 1**). The lower ST_100_ was used as a sublethal lower temperature in hardening assays.

**Table 1:** Expected survival at lower and upper temperatures in *C. cosyra*. Temperatures were calculated using the ‘dose.p’ function in R.

Proportional Survival	Calculated lower temperature (°C)	Standard Error	Calculated upper temperature (°C)	Standard Error
**0.1**	-10.95	0.63	43.13	54.33
**0.3**	-7.80	0.42	42.98	84.91
**0.5**	-5.83	0.34	42.88	104.11
**0.7**	-3.85	0.35	42.83	123.31
**0.9**	-0.70	0.51	42.63	153.89
**0.999**	10.28	1.42	42.10	260.59

### Experiment 2. Dietary effects on thermal tolerance and weight

**Figure 3:**
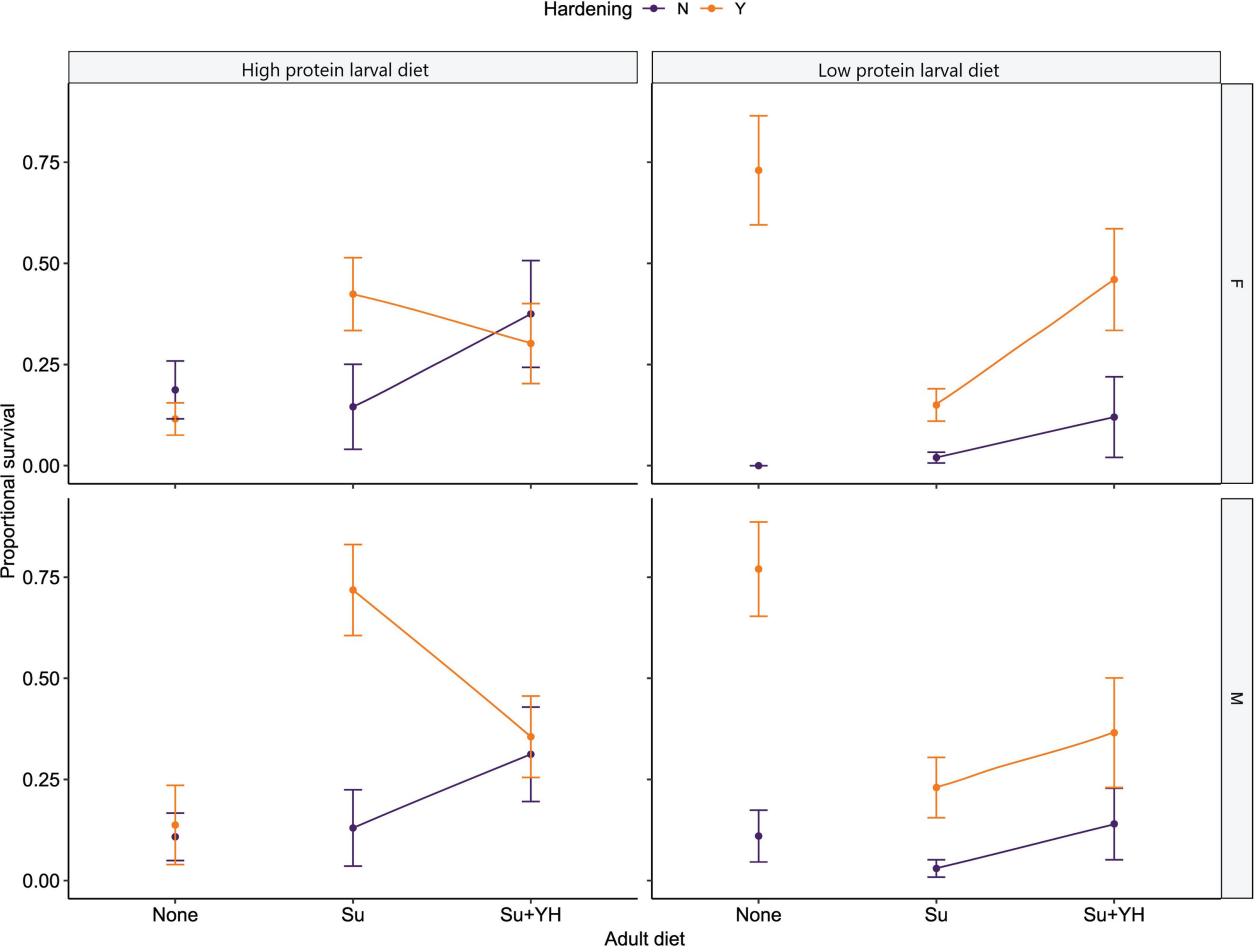
Proportional survival at -5.8°C of hardened or unhardened *C. cosyra* reared on either a low or high protein larval diet before being introduced to one of three adult dietary regimes. The dietary regimes are no adult diet (None), sugar (Su) and sugar plus yeast hydrolysate (Su+YH). Flies of the None dietary regime were tested on day of emergence (Day = 0) and remaining flies were split between the other two dietary regimes. The flies were then given 10 days to feed on the adult diets before being tested (Day = 10). Error bars represent the standard error of the mean.

Proportional survival of *C. cosyra* tested at -5.8°C was significantly affected by larval diet (χ^2^ = 27.88, df = 1, p < 0.001) as well as the nested effects of adult diet within larval diet (χ^2^ = 34.66, df = 4, p < 0.001), hardening within adult diet (χ^2^ = 210.44, df = 6, p < 0.001) and sex within hardening (χ^2^ = 42.59, df = 12, p < 0.001). When protein was restricted in either adult diet or larval diet, a brief exposure to sublethal lower temperatures prior to exposure to lower lethal temperatures boosted fly survival. For adults (males and females) reared from a high protein larval diet, lower survival was recorded for those that were both hardened and not fed compared with those that were both hardened and fed on a low-quality adult diet (Su). However, for adults reared from a low protein larval diet, higher survival was recorded for those that were both hardened and unfed compared with those that were both hardened and fed either with Su or Su+YH (**Figure 3**). Flies reared on a low protein larval diet that were denied access to an adult diet had a much higher proportional survival when compared with individuals with access to either of the adult diets, though flies with access to the protein rich Su+YH adult diet had a higher proportional survival than flies with access only to a protein poor Su adult diet (**Figure 3**). Furthermore, hardened Su fed male flies reared from a high larval protein diet had the highest proportional survival (~0.75) when compared with any other fly group apart from hardened male and female flies (both also around 0.75 proportional survival) reared on a low protein larval diet and denied access to an adult diet (**Figure 3**).

**Figure 4:**
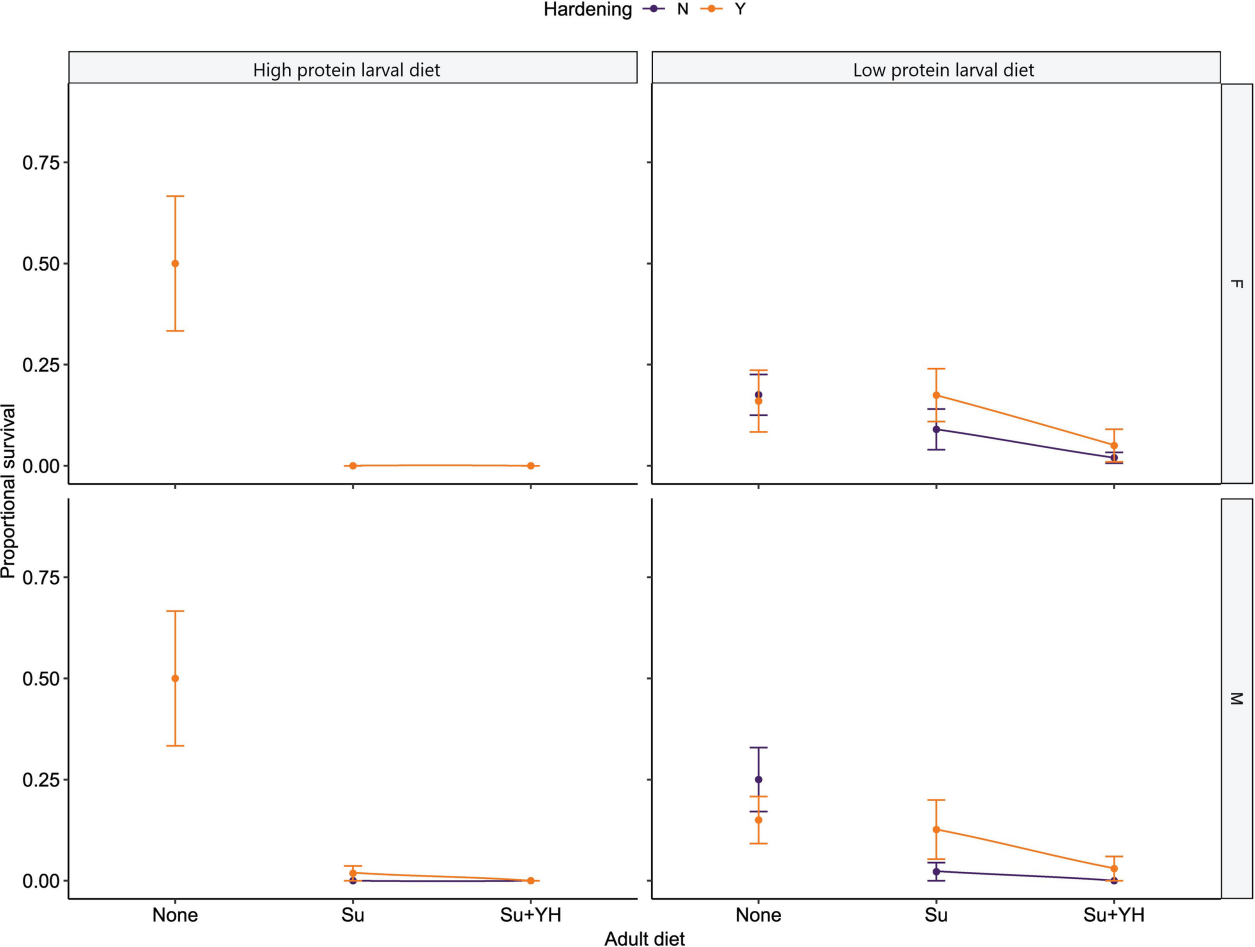
Proportional survival at 42.9°C of hardened or unhardened *C. cosyra* reared on either a low or high protein larval diet before being introduced to one of three adult dietary regimes. The dietary regimes are no adult diet (None), sugar (Su) and sugar plus yeast hydrolysate (Su+YH). Flies of the None dietary regime were tested on day of emergence (Day = 0) and remaining flies were split between the other two dietary regimes. The flies were then given 10 days to feed on the adult diets before being tested (Day = 10). Error bars represent the standard error of the mean.

Proportional survival of *C. cosyra* tested at 42.9°C was significantly affected by larval diet (χ^2^ = 23.61, df = 1, p < 0.001) as well as the nested effect of adult diet within larval diet (χ^2^ = 148.59, df = 4, p < 0.001). At high lethal temperatures, hardening and sex had no significant effect on the proportional survival of flies. At the high test temperature, both hardened and unhardened flies of both sexes reared on a high protein larval diet without access to adult diets had a higher proportional survival than flies reared on high protein larval diet with access to adult diets. In fact, the proportional survival of flies raised on a high protein larval diet and given access to an adult diet was essentially zero (**Figure 4**). As for low protein reared flies, the proportional survival for all three dietary combinations was mostly sub 0.25 with a great deal of overlap (**Figure 4**).

**Figure 5:**
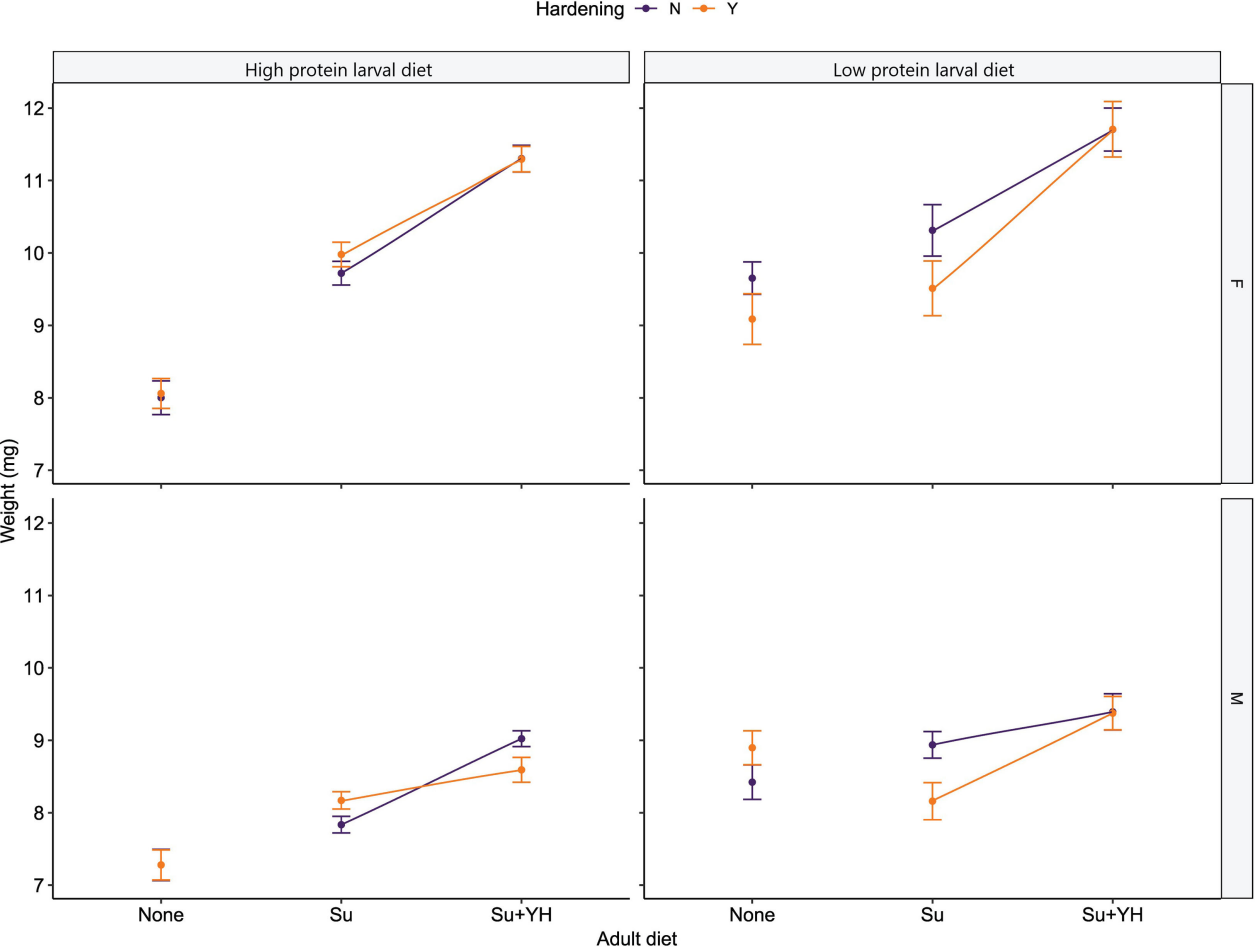
Weight of high and low protein reared *C. cosyra* on three adult dietary regimes. The dietary regimes are no adult diet (None), sugar (Su) and sugar plus yeast hydrolysate (Su+YH). Flies of the None dietary regime were tested on day of emergence (Day = 0), then split between the other two dietary regimes and tested 10 days after emergence (Day = 10) to allow time for feeding. Error bars represent mean standard error.

Weight was significantly affected by larval diet (χ^2^ = 23.66, df = 1, p < 0.001) as well as the nested effects of adult diet within larval diet (χ^2^ = 132.86, df = 4, p < 0.001), and sex within hardening (χ^2^ = 317.36, df = 12, p < 0.001). In general, males tended to be smaller than their female counterparts (**Figure 5**). Furthermore, flies without access to an adult diet were smaller than their sugar-fed counterparts which, in turn, were smaller than their sugar and yeast hydrolysate-fed counterparts (**Figure 5**). The exception being low protein reared and sugar-fed hardened male flies were lighter than their counterparts that were not hardened (**Figure 5**). However, the nested effect of hardening within adult diet within larval diet was not significant (χ^2^ = 8.95, df = 6, p = 0.18).

## Discussion

Newly emerged and hardened *C. cosyra* individuals were better able to survive low temperature extremes when compared with the other fly groups. When individuals reared on a low protein larval diet were cold hardened later in their adult life and after feeding on a low-quality adult diet, their ability to survive lower temperatures improved when compared with similar individuals that did not undergo hardening. However, survival was generally lower for hardened older flies than hardened newly emerged flies. This points to limitations in *C. cosyra* cold hardening ability, which could affect its distribution range. Physiological mechanisms resulting in rapid cold hardening include heat shock and cold stress protein production as well as the stockpiling of nutrients like glycerol, trehalose, polyhydric sugars, and alcohols (12, 13, 43–51). Survival of hardened *C. cosyra* flies fed on a sugar only (or carbohydrate rich) adult diet was significantly improved compared with flies not fed on an adult diet when reared on a high protein larval diet. For flies reared on a low protein larval diet, hardening improved proportional survival for all dietary combinations, with flies lacking adult diet access having the highest survival. Although not significant, there was a trend for flies fed only sugar to survive longer at low temperatures than those fed sugar and yeast. Similarly, a positive association between nutrient restriction and cold tolerance was observed in *D. melanogaster* and *D. ananassae* (Doleschall) (Diptera: Drosophilidae), where flies that were fed a carbohydrate rich diet had faster chill coma recovery times when compared with protein fed flies (20–22). For *Drosophila* spp, this was credited to an increase in fat content following carbohydrate intake (52–55). Greater lipid deposits could influence water loss by affecting either the quality or number of cuticular hydrocarbons (56). This may be important as nutritional state and hydration could regulate ion concentration *via* compensatory water intake, thus potentially aiding chill coma recovery and resistance to cold temperatures (22, 57). In *C. cosyra*, adult diet was previously not found to influence lipid reserves (28). As such, a mechanism other than metabolism of lipid reserves may be at play in influencing their hardening ability and tolerance to cold temperature.

At high temperatures, there was no significant phenotypic plasticity in *C. cosyra*. A protein rich adult diet was expected to have a significant impact on hardening and improve *C. cosyra* survival at ULT as seen in the tropical drosophilid flies *D. melanogaster* and *D. ananassae* (19, 20). The underlying physiological mechanisms linked to rapid heat hardening involve heat shock protein production (45, 46, 51). The precipitous drop in survival of *C. cosyra* at high temperatures implies that the species is already living near optimal temperature. Plasticity in upper thermal limits for species living close to their upper lethal temperatures is unlikely to effectively mitigate effects of increasing global temperature (1, 2, 58, 59). This could explain the lack of any significant hardening results in *C. cosyra* at ULT. Age could also be a confounding factor as it is known to cause a massive decrease in heat shock resistance in holometabolous insects (60–67). Insects that feed during the adult stage usually do so to improve energy supply in preparation for reproduction (50). This could imply a possible trade-off between reproduction, nutritional prioritization, and thermal tolerance. Adult insects also use nutrients for somatic maintenance, which could also lead to a trade-off with heat shock resistance (68). In comparison with lethal temperatures recorded for other congeneric species such as *C. capitata* and *C. rosa* (13), we found using the same methodology in this study a higher ULT and a slightly lower LLT for *C. cosyra*. Sex did not significantly affect thermal tolerance of *C. cosyra* at high temperatures and matches similar findings in *C. capitata* and *C. rosa.* However, sex did appear to have a significant effect on thermal tolerance under LLT conditions when nested within larval diet, adult diet and hardening, which was unexpected. When thermal tolerance of other tephritid flies has been measured, sex usually has little effect (13, 22, 69, 70).

Nutritional status has been linked to thermal tolerance ability in mathematical models and various empirical studies (22, 41, 71–73). Body weight has generally been assumed to predict resistance to stressors. This is because larger body weight is linked to larger nutritional reserves, which play an important role in insect life history traits like growth rate and resistance to various stressors such as heat or cold (22, 50). However, this study found no significant interaction between diet and hardening regarding fly body weight. This could imply that body weight is not an ideal indicator of thermal stress tolerance in *C. cosyra*.

*Ceratitis cosyra* is a sub Saharan pest that affects much of Africa, and under a changing climate it is important to understand how it may respond to better prepare for either its spread or introduction into regions where susceptible hosts are present (74). The *C. cosyra* population used in this study was originally sourced from the northern Tshwane Metropolitan Municipality in the Gauteng province, which is located within the African savannah biome (75). This biome is often subjected to sub-zero temperatures (76, 77). The ability of *C. cosyra* to improve their cold tolerance indicates that individuals of the species can survive in environments of extreme temperature variability, especially lower temperatures. Our results indicate that this could possibly be achieved by *C. cosyra* feeding on a protein rich adult diet to compensate for feeding on a low protein mango or marula larval diet. A diet rich in amino acids is, however, limited in natural environments (78–80). Also, the literature, shows that adult dipterans that feed on a protein rich adult diet often prioritize reproductive capability to the detriment of cold tolerance (20, 50, 81, 82). The broad basal temperature range that we observed, as well as the species’ observed phenotypic plasticity in thermal tolerance after brief exposure to sublethal cold temperatures could also contribute to survival at lower temperatures. However, perhaps an equally important determinant of the geographical range of *C. cosyra* is the distribution of its main indigenous host plant, the marula (83). The effects of temperature on its host plants may indirectly influence the distribution of *C. cosyra*. Climate change could cause a species distribution shift by shifting host plant distribution (7, 10, 83).

The effect of changing climate on overwintering should also be considered. Insects use environmental cues such as photoperiod and temperature (and their interaction) to enter a dormancy period that causes arrestment of development, nutrient reserve storage, metabolic decrease, and an increase in cold resistance (84, 85). This means that increasing night length (or decreasing day length) coupled with decreasing autumn temperatures can induce diapause in flies such as *Calliphora vicina* (Robineau-Desvoidy) (Diptera: Calliphoridae), *Sarcophaga crassipalpis* (Macquart) (Diptera: Sarcophagidae), and *C. capitata* as a mechanism to survive the winter (86–88). Evidence suggests that changing climatic conditions are impacting many insect species’ ability to overwinter (89). Evidence suggests that *C. capitata* is unable to overwinter in Germany and current populations are transient in nature due to reinvasions caused by fruit importations (88). However, future climate scenarios (2030 and 2050) show that changing temperature could result in the successful establishment of *C. capitata* populations in Germany (90). Future modelling for habitat suitability has also been done for *C. cosyra* on the African continent, indicating an increase in habitat suitability when taking temperature into consideration (7). Habitat suitability improvements could also result in an improved ability to overwinter in those areas, allowing for establishment of populations in areas where previously absent. Conversely, changing temperatures and photoperiod could result in species feeding during the cold of winter, which is not ideal for winter survival (81, 91, 92).

## Conclusions

Diet and hardening influenced thermal tolerance of *C. cosyra*, while sex only seemed to affect survival at LLT. The nutrition and hardening effect on thermal tolerance was temperature dependent. At low temperatures, nutrient restriction combined with rapid cold hardening enhanced survival. At high temperatures, there was little evidence for hardening, and survival of adult fed flies was lower. This might be caused by the influence of age or mating on heat tolerance. *Ceratitis cosyra* ability to both compensate for a low protein larval diet and undergo rapid cold hardening after a brief exposure to sublethal cold temperatures even when the subsequent adult fed on low protein diets indicates that individuals of the species have improved survivability in extreme temperature variable environments, particularly at low temperatures. However, there appears to be limitations to *C. cosyra* ability to cold harden and the species may be more at risk from chronic effects than from exposure to acute thermal stress.

## Data availability statement

The original contributions presented in the study are publicly available. This data can be found here: https://doi.org/10.25403/UPresearchdata.22262965.

## Author contributions

The experiment was conceived and designed by CW. DP performed the experimentation and collected the data. DP ran the data analyses. DP wrote the manuscript. All authors contributed to the article and approved the submitted version.
