# Supplementary material for: Larval and adult diet affect phenotypic plasticity in thermal tolerance of the marula fly, Ceratitis cosyra (Walker) (Diptera: Tephritidae)
Source: Front Insect Sci. 2023 Mar 28;3:1122161. doi: 10.3389/finsc.2023.1122161 (PMC10926529; doi:10.3389/finsc.2023.1122161)
Supplement: Supplementary file 1 [file DataSheet_1.zip › Appendix 1.DOCX]

**Appendix 1. Estimated relative humidity within test vials at temperatures used in Experiments 1 and 2.**

Due to the small size of the containers used for our experiments, we were unable to utilize equipment to directly measure the relative humidity (RH) experienced by flies in each experiment. However, using the temperature of the climate room used for rearing, maintenance, and the 30 min rest period before the temperature assays took place, as well as the average humidity of the climate room (50%), we estimated RH during each temperature assay using a formula based on work done by Lawrence (2005) and Alduchov & Eskridge (1996):


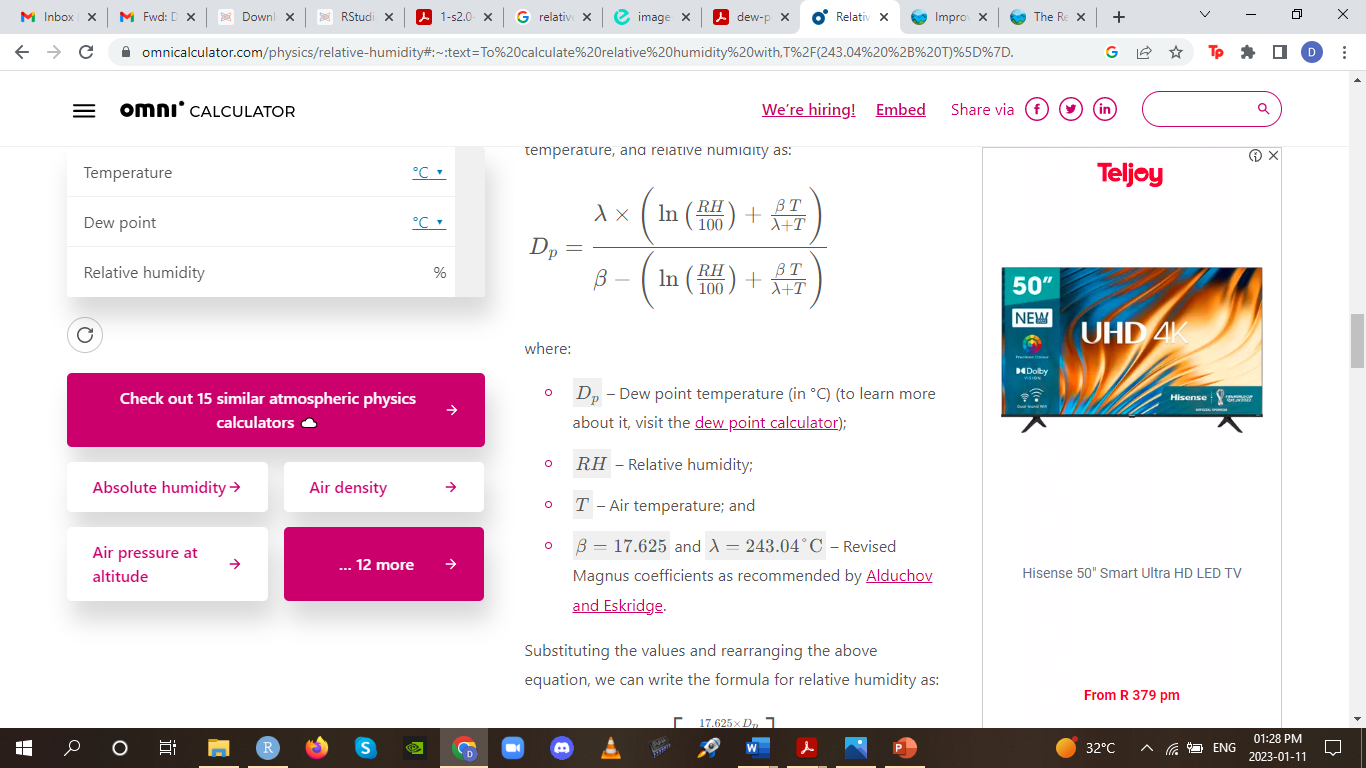


Where: *Dp* is dew point temperature (in °C)

- *RH* is relative humidity
- *T* is air temperature
- *β* = 17.625 and *λ* = 243.04°C – Revised Magnus coefficients as recommended by Alduchov & Eskridge (1996)

Rewritten as a formula for RH:


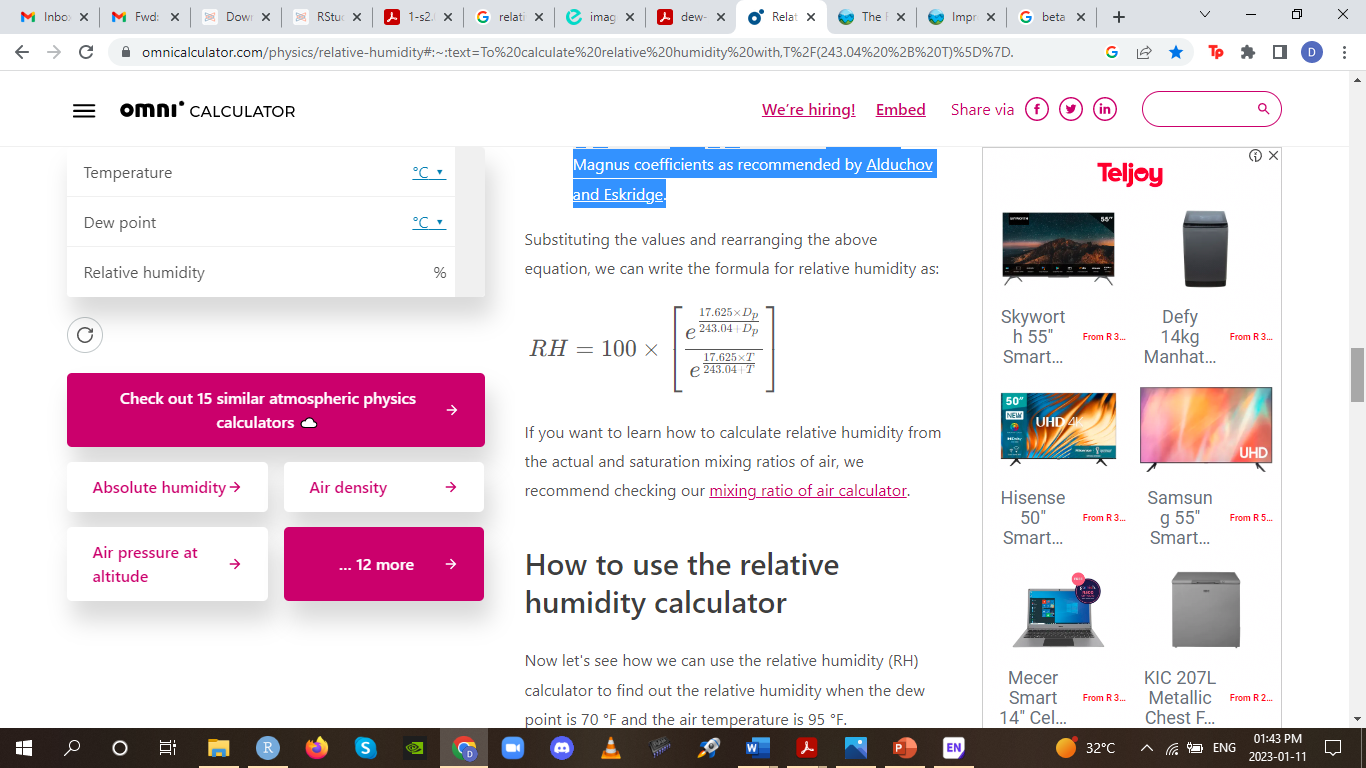


The RH of the vials in Experiment 1 and 2 are as follows (values in red are calculated values that were corrected to 100% RH):

**Experiment 1**

52.0°C = 9.66%

48.1°C = 11.73%

43.4°C = 14.94%

40.3°C = 17.59%

35.7°C = 22.58%

32.1°C = 27.61%

28.1°C = 34.73%

14.0°C = 82.63%

10.1°C = 100% (106.81%)

6.4°C = 100% (137.30%)

3.2°C = 100% (171.60%)

-2.2°C = 100% (253.50%)

-5.9°C = 100% (334.56%)

-11.9°C = 100% (534.7%)

**EXP 2**.

42.9°C = 15.33%

35.0°C = 23.47%

10.3°C = 100% (105.4%)

-5.8°C = 100% (332%)

It is important to note that *C. cosyra* has a high desiccation resistance measured in days and not hours, as noted in Weldon *et al.* (2019). RH and water stress are unlikely to have any effect during temperature assays as short as 2-3 hours.

**References**

Alduchov, O.A. & Eskridge, R.E. 1996. Improved magnus form approximation of saturation vapor pressure. *Journal of Applied Meteorology and Climatology* **35**: 601-609.

Lawrence, M.G. 2005. The relationship between relative humidity and the dewpoint temperature in moist air: A simple conversion and applications. *Bulletin of the American Meteorological Society* **86**: 225-234.

Weldon, C.W., Mnguni, S., Demares, F., Rand, E.E.d., Malod, K., Manrakhan, A. & Nicolson, S.W. 2019. Adult diet does not compensate for impact of a poor larval diet on stress resistance in a tephritid fruit fly. *Journal of Experimental Biology* **222**: 192534.
